# Supplementary material for: Monkey plays Pac-Man with compositional strategies and hierarchical decision-making
Source: eLife. 2022 Mar 14;11:e74500. doi: 10.7554/eLife.74500 (PMC8963886; doi:10.7554/eLife.74500)
Supplement: Transparent reporting form [file elife-74500-transrepform1.docx]

***eLife’s* transparent reporting form**

We encourage authors to provide detailed information *within their submission* to facilitate the interpretation and replication of experiments. Authors can upload supporting documentation to indicate the use of appropriate reporting guidelines for health-related research (see [EQUATOR Network](http://www.equator-network.org/%20)), life science research (see the [BioSharing Information Resource](https://biosharing.org/)), or the [ARRIVE guidelines](http://www.plosbiology.org/article/info:doi/10.1371/journal.pbio.1000412) for reporting work involving animal research. Where applicable, authors should refer to any relevant reporting standards documents in this form.

If you have any questions, please consult our Journal Policies and/or contact us: [editorial@elifesciences.org](mailto:editorial@elifesciences.org).

**Sample-size estimation**

- You should state whether an appropriate sample size was computed when the study was being designed
- You should state the statistical method of sample size computation and any required assumptions
- If no explicit power analysis was used, you should describe how you decided what sample (replicate) size (number) to use

Please outline where this information can be found within the submission (e.g., sections or figure legends), or explain why this information doesn’t apply to your submission:

No power calculations to determine sample size in advance were performed.

The behavior analyses include data from 74 testing sessions for two monkeys after their performance reached a reasonable level. We recorded the joystick movements, eye movements, and pupil sizes of the animals during the game. On average, the animals completed 33±9 (mean±SE) games in each session and each game took them 4.9±1.8 attempts. In total, we recorded 3217 games, 15772 rounds, and 899381 joystick movements.

This information can be found in Results ‘The Pac-Man Game’, Method ‘Behavioral Data Recording and Preprocessing’ and Figure S2.

**Replicates**

- You should report how often each experiment was performed
- You should include a definition of biological versus technical replication
- The data obtained should be provided and sufficient information should be provided to indicate the number of independent biological and/or technical replicates
- If you encountered any outliers, you should describe how these were handled
- Criteria for exclusion/inclusion of data should be clearly stated
- High-throughput sequence data should be uploaded before submission, with a private link for reviewers provided (these are available from both GEO and ArrayExpress)

Please outline where this information can be found within the submission (e.g., sections or figure legends), or explain why this information doesn’t apply to your submission:

Biological replication is defined as the number of subjects performed the same task and the number of sessions and trials performed by each subject.

There are no technical replicates included.

Two healthy, male rhesus macaque (Macaca mulatta) monkeys (O and P) weighting 6-7 kg, performed Pac-Man task. We recorded a total of 74 sessions from both monkeys, for a total of 3217 games, 15772 rounds, and 899381 joystick movements. This information can be found in Method ‘Subjects and Materials’, ‘Behavioral Data Recording and Preprocessing’ and Figure S2.

When studying the pupil dilation of monkeys during the game-play, we excluded data that were 3 standard deviations away from the mean. This information can be found in Method ‘Eye Movement Analysis’.

**Statistical reporting**

- Statistical analysis methods should be described and justified
- Raw data should be presented in figures whenever informative to do so (typically when N per group is less than 10)
- For each experiment, you should identify the statistical tests used, exact values of N, definitions of center, methods of multiple test correction, and dispersion and precision measures (e.g., mean, median, SD, SEM, confidence intervals; and, for the major substantive results, a measure of effect size (e.g., Pearson's r, Cohen's d)
- Report exact p-values wherever possible alongside the summary statistics and 95% confidence intervals. These should be reported for all key questions and not only when the p-value is less than 0.05.

Please outline where this information can be found within the submission (e.g., sections or figure legends), or explain why this information doesn’t apply to your submission:

We used mean to define the center value. We used standard error and standard deviation as precision measures.

This information can be found in Figure 1B-C, 2B-D, 3A-B, 3F, 5C-D, 6C-D, S2A-L, S3A-D, S4ABEFG, S5ABEF, S6B-H, S7B-H, S8A-H legends.

Two-sample t-test is used to compute p-values, determining the significance level of the similarity of two group of data.

This information can be found in Figure 3F, 5B-D, 6A-D, S6B-H, S7B-H, S8A-H legends.

(For large datasets, or papers with a very large number of statistical tests, you may upload a single table file with tests, Ns, etc., with reference to sections in the manuscript.)

**Group allocation**

- Indicate how samples were allocated into experimental groups (in the case of clinical studies, please specify allocation to treatment method); if randomization was used, please also state if restricted randomization was applied
- Indicate if masking was used during group allocation, data collection and/or data analysis

Please outline where this information can be found within the submission (e.g., sections or figure legends), or explain why this information doesn’t apply to your submission:

This information is not available in the Pac-Man task discussed in the paper.

**Additional data files (“source data”)**

- We encourage you to upload relevant additional data files, such as numerical data that are represented as a graph in a figure, or as a summary table
- Where provided, these should be in the most useful format, and they can be uploaded as “Source data” files linked to a main figure or table
- Include model definition files including the full list of parameters used
- Include code used for data analysis (e.g., R, MatLab)
- Avoid stating that data files are “available upon request”

Please indicate the figures or tables for which source data files have been provided:

The sample data that supports the findings of this study and all custom code used for analysis and figure generation will be viewable and available for download from github.com/superr90/Monkey_PacMan.
